# Dynamics Management of Intermediate Water Storage in an Air-Breathing Single-Cell Membrane Electrode Assembly

**DOI:** 10.3390/membranes14010004

**Published:** 2023-12-22

**Authors:** Avinash Kumar, Alex Schechter, Idit Avrahami

**Affiliations:** 1 Department of Mechanical Engineering and Mechatronics, Ariel University, Ariel 40700, Israel; avinash481@gmail.com; 2 Department of Chemical Sciences, Ariel University, Ariel 40700, Israel; salex@ariel.ac.il

**Keywords:** hydrogen fuel cell, proton exchange membrane, humidity, water content, inflatable hydrogen storage system, anode self-humidification, water storage, air breathing, membrane electrode assembly, hydration state

## Abstract

In air-breathing proton exchange membrane fuel cells (Air PEM FCs), a high rate of water evaporation from the cathode might influence the resistance of the membrane electrode assembly (MEA), which is highly dependent on the water content of the Nafion membrane. We propose a dead-end hydrogen anode as a means of intermediate storage of water/humidity for self-humidification of the membrane. Such an inflatable bag integrated with a single lightweight MEA FC has the potential in blimp applications for anode self-humidification. A dynamic numerical water balance model, validated by experimental measurements, is derived to predict the effect of MEA configuration, and the membrane’s hydration state and water transfer rate at the anode on MEA resistance and performance. The experimental setup included humidity measurements, and polarization and electrochemical impedance spectroscopy tests to quantify the effect of membrane hydration on its resistance in a lightweight MEA (12 g) integrated with an inflatable dead-end hydrogen storage bag. Varying current densities (5, 10, and 15 mA/cm^2^) and cathode humidity levels (20, 50, and 80%) were examined and compared with the numerical results. The validated model predicts that the hydration state of the membrane and water transfer rate at the anode can be increased by using a thin membrane and thicker gas diffusion layer.

## 1. Introduction

Proton-exchange-membrane (PEM) fuel cells (FCs) have come out as a promising alternative to grid-based power generation as they can produce clean energy by directly converting the chemical energy of the fuel (hydrogen) into electrical power and have a relatively low operating temperature compared to other types of FCs [1,2]. PEM FCs consist of units of a membrane electrode assembly (MEA), each of which includes two electrodes called a cathode and an anode separated by an electrolyte. Over the years, it has been proposed to reduce the complexity and weight of PEM FCs by exposing the cathode to free convection ambient airflow to supply oxygen to its cathode and to have a simpler lightweight design [1,3,4,5]. This transition to an air-breathing cathode PEM FC allows for a lower weight but poses a challenge in terms of water management.

The electrolyte membrane is the pivotal component of the air-breathing PEM FC, which is responsible for the ion transfer between the anode and the cathode in order to maintain the charge balance and allow for power output. In addition, the membrane prevents the cross-mixing of the gas between the anode and the cathode. A Nafion^TM^ membrane is the most commonly used electrolyte due to its good mechanical properties, high proton conductivity, chemical stability, and low internal resistance [6,7]. Hydrogen-based air-breathing proton-exchange-membrane fuel cells (Air PEM FC) mostly use Nafion^TM^ membranes, which are perfluorosulfonic acid (PFSA) membranes with perfluorinated backbones and sulfonic acid as the terminal group.

The gas diffusion layer (GDL) is another significant component of the MEA. It is a microporous layer of carbon fiber or cloth placed in contact with the catalyst layer on both sides of the membrane. The GDL is a critical component of an FC, as it facilitates the transport of reactants and products and plays a key role in water and thermal management [8,9,10].

One of the major disadvantages of Air PEM FCs is that the water evaporation from the cathode to the ambient atmosphere is high, thereby influencing the resistance of the Nafion membrane and leading to unstable power output [11,12,13,14,15]. Therefore, sufficient humidity of the membrane must be maintained during the fuel cell operation to improve the power output and prolong its service life. Several humidification methods have been suggested, including internal humidification [16], external humidification [17], and self-humidification [18,19]. Internal methods, such as water stored in cathode end plates, and external techniques, such as bubble humidifiers, are more effective but require heavier and more complex monitoring and control subcomponents. Water injection to electrodes in direct or indirect modes has improved FC performance [20,21]. Self-humidification methods such as adding hydrophilic nanoparticles to the membrane [22,23,24] or catalyst layer [25,26,27,28] and using a double gas diffusion layer [18] have been effective for low-power applications. Still, they are also significantly influenced by the operating conditions. In a worst-case scenario, when the Air PEM FC is required to operate in a dry ambient condition, the self-humidification method must rely solely on water produced electrochemically at the cathode.

To ensure high FC efficiency, the MEA units are stacked in rigid fixtures of heavy bipolar plates to allow sufficient sealing pressure and flow-field channels. In very small and light applications, where a single MEA is sufficient, the heavy rigid structure can be avoided and the super lightweight MEA may supply extremely high energy densities for extended operation durations at a relatively low power and voltage (0.5–0.7 V). Such applications may include small sensors, tracking systems, metrological stations, miniature drones, or blimps that may benefit from an extremely high energy density at a low power [29,30].

In such applications, where a single MEA is used as an FC, water balance over the MEA is critical for obtaining optimal results. On one hand, the efficient use of the FC at high current densities tends to dehydrate the membrane, and on the other hand, the accumulation of water produced at the cathode could induce electrode flooding, which impedes gas diffusion to the electrodes [31,32,33,34]. Specifically, the Nafion^TM^ membrane’s conductivity properties are strongly affected by its humidification/hydration state, i.e., the water content of the membrane, which is defined by the amount of water absorbed on the sulfonic sites and the temperature of the fuel cell [35,36]. Therefore, understanding the transport of water produced at the cathode across the MEA during FC operation and the suitable selection of membrane and GDL characteristics are essential for developing suitable water management strategies for the large-scale commercialization of single MEA applications.

In the MEA, current flow is associated with the drag of water molecules from the anode to the cathode side, a phenomenon known as electro-osmotic drag (EOD) transport, which depends on the water content of the membrane [37]. These dragged water molecules, combined with water produced at the cathode, accumulate on the cathode side of the membrane. This accumulation creates a water content gradient within the membrane, leading to the back-diffusion of water molecules in the opposite direction [38]. The net transport of water across the membrane is influenced by the water concentration at the electrodes and other factors such as temperature, pressure, membrane water content, membrane thickness, gas diffusion layer (GDL), and the humidity of the reactant gases [39]. Therefore, achieving a balanced water management condition is essential to controlling the desired direction of water transport across the MEA during FC operation to ensure that the cathode is not flooded due to water accumulation or that the anode does not become too dry due to strong electro-osmotic drag. Maintaining this balance is crucial for an effective self-humidification design to prevent membrane damage and ensure a reliable power output even in severe temperature and environmental conditions [27,39,40,41].

In this study, we propose a dead-end hydrogen anode chamber as a means of intermediate water storage to maintain the hydration level of the membrane and the hydrogen gas feed stream. A compliant dead-end hydrogen storage chamber at the anode, such as a balloon, a local pocket, or a small container at ambient pressure, is utilized to trap the water produced at the cathode traveling to the anode side. This low-cost and lightweight self-humidification method can be applied in small applications, such as a local pocket or a small container at ambient pressure, to keep the membrane hydrated under dry ambient conditions—for example, a hydrogen blimp powered by Air PEM FCs [40,41].

To illustrate the benefits of the suggested dead-end anode chamber, we developed a simple water balance analytical model to describe the transfer of water in an air-open PEM FC integrated with a dead-ended inflatable hydrogen bag at the anode. We constructed an experimental setup to validate this model. The model allows us to predict the impact of variables such as GDL thickness, membrane thickness, current density, cathode humidity, and bag volume.

## 2. Materials and Methods

**Figure 1:**
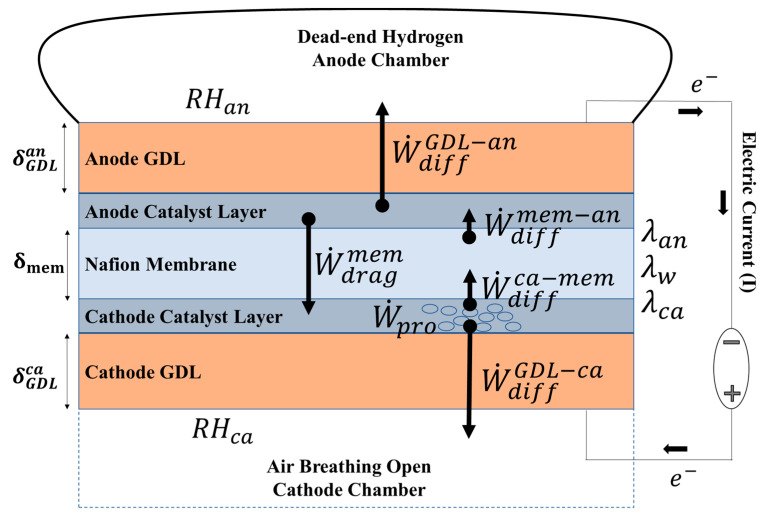
A schematic diagram illustrating the numerical model of water transfer across the membrane electrode assembly (MEA), which includes anode and cathode gas chambers, gas diffusion layers (GDL-anode and cathode), catalyst layers, and a proton exchange membrane (Nafion^TM^). The anode is confined by a closed compartment filled with hydrogen gas.

Figure 1 shows a schematic diagram of the water balance model, including a membrane electrode assembly (MEA) placed between the ambient cathode and a dead-end hydrogen storage bag (anode). The MEA is composed of a Nafion membrane, anode and cathode catalytic layers, and anode and cathode GDLs.

As shown in Figure 1, the electrochemical process results in the production of water at the cathode (${\dot{W}}_{pro}$) as per the following electrochemical reaction mechanism.

Anode (Oxidation): H_2_ → 2 H^+^ + 2*e*^− ^   E^0^_anode_ = 0

Cathode (Reduction): ½O_2_ + 2*e*^−^ + 2H^+^ → H_2_O   E^0^_cathode_ = 1.229 V

Overall reaction:  H_2_ + ½O_2_ → H_2_O   E^0^_cell_ = 1.229 V

Two primary mechanisms of water transport work across the membrane at the same time: (1) the electro-osmotic drag (${\dot{W}}_{drag}^{mem}$) of water molecules from the anode to the cathode, and (2) back-diffusion flow (${\dot{W}}_{diff}^{ca-mem}$) of water from the cathode to the membrane. A part of the produced water is evaporated from the cathode through the cathode GDL (${\dot{W}}_{diff}^{GDL-ca}$). The remaining portion is absorbed into the membrane and subsequently trapped in the anode chamber (${\dot{W}}_{diff}^{GDL-an}$). Cathode humidity (${RH}_{ca}$) is considered a fixed value determined by the ambient condition and the anode humidity (${RH}_{an}$) is calculated as a function of time.

The hydration level of the membrane ($\lambda_{w}$) is defined by the molar ratio of water and the sulfonic acid groups and strongly influences the ionic resistance of the membrane. A maximum value of $\lambda_{w}=22$ is reported with liquid water saturation [42,43]. In practice, this value cannot be achieved in an FC MEA due to the hot-pressing process that leads to the shrinking of the hydrophilic channels. The water content of the membrane at the interface of the membrane–cathode ($\lambda_{ca}$) and membrane–anode ($\lambda_{an}$) determines the resultant water content of the membrane ($\lambda_{w}$), which depends on water transfer dynamics across the MEA.

A numerical model of the water balance is derived as detailed below to predict the effect of changes in current density (*I*), ambient cathode relative humidity (${RH}_{ca}$), GDL thickness ($\delta_{GDL}^{an}=\delta_{GDL}^{ca}$), and membrane thickness ($\delta_{mem}$) on anode humidity (${RH}_{an}$), the water content of the membrane ($\lambda_{w}$), and the ionic resistance of the membrane.

### 2.1. Transient Water Balance Model of the Membrane Electrode Assembly (MEA)

The main objective of the model is to predict the general trend in water transfer over a simplified representation of the membrane. The model assumes one-dimensional water transfer, as water transport occurs perpendicular to the membrane. Additionally, an isothermal condition is considered due to the low working temperature of an air-breathing single MEA (20–40 °C) compared to a standard FC (50–90 °C). The low working temperature of air-breathing FCs is mainly caused by the high activation energy of the oxygen reduction reaction at the cathode due to weak natural convective mass transfer that reduces the current density [44,45]. Moreover, the direct contact of the electrodes with the ambient air in the single air-breathing MEA configuration, along with the relatively low power, maintains a low-temperature regime. Thus, modeling the effect of temperature rise is out of the scope of this study. Water diffusion from the anode to the membrane is neglected due to the higher concentration of produced water at the cathode. Gas crossover across the membrane is also neglected.

A transient mass balance of water at the cathode catalyst layer, anode catalyst layer, and across the membrane shown in Figure 1 can be represented by Equations (1)–(3). Water transport from cathode to anode is considered positive.

(1) $${\dot{W}}_{pro}={\dot{W}}_{diff}^{GDL-ca}+{\dot{W}}_{diff}^{ca-mem}-{\dot{W}}_{drag}^{mem},$$

(2) $${\dot{W}}_{diff}^{GDL-an}={\dot{W}}_{diff}^{mem-an}-{\dot{W}}_{drag}^{mem},$$

(3) $$\frac{A_{mem}{\;\delta }_{mem}\rho_{mem,dry}}{EW}\left(\frac{d\lambda_{w}}{dt}\right)={\dot{W}}_{diff}^{ca-mem}-{\dot{W}}_{diff}^{mem-an}-{\dot{W}}_{drag}^{mem},$$

where $A_{mem}$ is the active area of the MEA, $\delta_{mem}$ is the membrane thickness, $\frac{d\lambda_{w}}{dt}$ is the change in membrane water content with time (*t*), $\rho_{mem,dry}$ is the membrane’s dry density, and EW is the equivalent weight of the membrane. The terms in Equations (1)–(3) can be calculated based on the standard relationship between the variables as follows:

Rate of water production at the cathode,

(4) $${\dot{W}}_{pro}\left[\frac{kg}{s}\right]=\frac{A_{mem\;}M_{w}I\;}{2F},$$

where the molecular weight of water vapor $M_{w}=0.018[\frac{kg}{mol}]$ and Faraday’s constant $F=96485[\frac{sA}{mol}]$. The current density (*I*) is an operating variable.

(5) $${\dot{W}}_{diff}^{GDL-i}\left[\frac{kg}{s}\right]=-\frac{A_{mem}D_{GDL}^{i}}{\delta_{GDL}^{i}}(C_{w}^{i}-C_{i}),$$

where ‘*i*’ represents the cathode (ca) or anode (an). $D_{GDL}^{i}$ and $\delta_{GDL}^{i}$ are the diffusion coefficient and thickness of the cathode/anode GDL. ‘$C_{w}^{i}$’ and ‘$C_{i}$’ are the molar concentrations of water vapor at the two membrane–electrode (cathode/anode) interfaces and in the ambient air, respectively, which are defined as:

(6) $$C_{w}^{i}=\frac{\rho_{mem,dry}}{EW}\lambda_{i},$$

(7) $$C_{i}=\frac{M_{w}\;{RH}_{i}\;P_{sat\;}}{R\;T},$$

where $\rho_{mem,dry}=2000\;[\frac{kg}{m^{3}}]$, $EW=1.1\left[\frac{kg}{mol}\right]$, the temperature of anode and cathode chambers is $T=295\left[K\right]$, and the gas constant is $R=8.314[\frac{{kgm}^{2}}{s^{2}Kmol}$]. Here, the water concentration at the surface of the anode and cathode GDLs is assumed to be equal to the chamber concentration for the described air-breathing dead-end anode system, and water is considered to be present in the vapor phase. *P_sat_* represents the saturation pressure of water vapor and can be calculated as [46,47]:

(8) $$P_{sat}\left[atm\right]=10^{-2.1794+0.02953\left(T-273.15\right)-9.1837\times 10^{-5}{\left(T-273.15\right)}^{2}+1.14454\times 10^{-7}{\left(T-273.15\right)}^{3}},$$

The ambient cathode humidity (${RH}_{ca}$) is fixed, and a measure of transient anode chamber humidity (${RH}_{an}$) can be expressed as:

(9) $${RH}_{an}(t)=\frac{\int_{0}^{t}{\dot{W}}_{diff}^{GDL-an}dt}{\left(\rho_{H_{2}}V_{b}+\int_{0}^{t}{\dot{W}}_{diff}^{GDL-an}dt\right)},$$

The volume (*V_b_*) of the inflatable anode bag can be calculated as,

(10) $$V_{b}=V_{0}-\frac{A_{mem\;}M_{H_{2}}\;I}{2F.\;\;\rho_{H_{2}}}\;t,$$

where $\rho_{H_{2}}$ = 0.08 [$\frac{kg}{m^{3}}$] is the density of hydrogen gas, $V_{0}$ is the initial volume of the hydrogen bag, and $M_{H_{2}}$ = 0.002 [$\frac{kg}{mole}$] is the molar mass of hydrogen gas.

The rate of water transfer from the (cathode/anode) membrane interface surface to the membrane is defined as

(11) $${\dot{W}}_{diff}^{i-mem}\left[\frac{kg}{s}\right]=\frac{A_{mem}\;D_{w}\;\rho_{mem,dry}\;\;\left|\lambda_{i}-\lambda_{w}\right|}{\delta_{mem}},$$

where $D_{w}$ is the water diffusivity relation, derived from experimental data in the membrane, and can be expressed as [48]

(12) $$D_{w}=\left\{\begin{array}{c} 3.1\times 10^{-7}\lambda_{w}\;\left(e^{0.28\lambda_{w}}-1\right)\;e^{\left(-2346/T\right)},\;\;0\leq \lambda_{w}<3\\ 4.17\times 10^{-8}\lambda_{w}\;\left({161e}^{-\lambda_{w}}+1\right)\;e^{\left(-2346/T\right)},\;\;\lambda_{w}\geq 3 \end{array}\right..$$

The drag of water molecules due to proton movement,

(13) $${\dot{W}}_{drag}^{mem}\left[\frac{kg}{s}\right]=\frac{A_{mem}\;\lambda_{w}\;I}{22\;F}\;n_{d\;}M_{w},$$

where the electro-osmotic drag coefficient $n_{d}$ is defined as the number of water molecules dragged from anode to cathode per proton and can be considered to vary as per water content or a constant. In this work, it was assumed to be a constant value ($n_{d\;}$ = 1) [37,46,49,50,51].

The electrolyte Nafion membrane conductivity ($\sigma_{mem}$) is an important property that depends on the water content of the membrane [46,52,53,54]:

(14) $$\sigma_{mem}=\left(0.514\lambda_{w}-0.326\right)e^{\left[1268(\frac{1}{303}-\frac{1}{T})\right]},$$

The membrane ionic resistance can be determined as

(15) $$R_{mem}=\frac{1}{\sigma_{mem}}$$

Table 1 summarizes the base parameters used in the model study unless stated otherwise.

The transient water balance Equations (1)–(3) are solved using MATLAB as per the above relationships to estimate the dynamic water content of the membrane ($\lambda_{w}$), anode humidity (${RH}_{an}$), and resistance of the membrane ($R_{mem}$).

### 2.2. Experimental Setup for Model Validation

**Figure 2:**
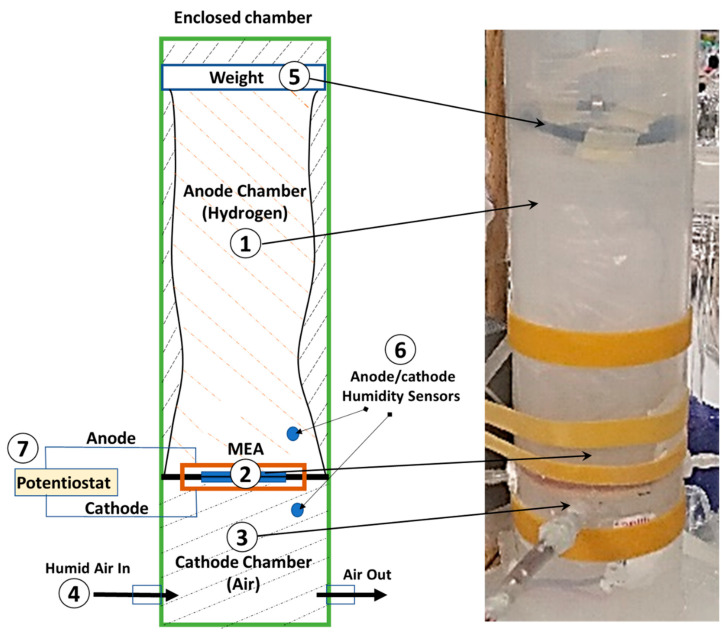
The experimental setup (a schematic illustration and a photo), including a cylinder with an inflatable dead-end hydrogen storage bag (1) connected to a lightweight MEA (2), a cathode chamber (3) with ports for humid airflow (4), a weight (5), humidity sensors (6), and a Potentiostat (7).

An experimental setup was designed to validate the model, as shown in Figure 2.

The setup consists of a cylindrical inflatable plastic bag (#1 in the figure) with a diameter of 6 cm and a length of 20 cm, serving as the hydrogen storage/supply chamber (anode). This plastic bag is supported by a rigid cylinder (ID = 6 cm and OD = 8 cm). A small flat weight (#5 in the figure) of 5 g is placed on top of the bag to keep the pressure constant and to facilitate easy measurements of the consumed hydrogen volume. It maintains a modest pressure difference between the anode and cathode sides to minimize the influence of water movement over the membrane due to the pressure gradient.

An MEA (#2 in the figure) with a catalyst-coated (0.5 mg/cm^2^ Pt-C) PEM (Nafion^TM^ N212, purchased from Fuelcellstore) with an area of $A_{mem}=2\times 2\;cm^{2}$ and a thickness of $\delta_{mem}=50\;µm\;$was prepared in our lab according to previously published protocols as described [55,56,57]. Catalyst ink was prepared by mixing 50 wt.% Pt/C in distilled water (18.2 MΩ cm), 5 wt.% Nafion solution (LQ 1105–1100 EW purchased from Ion Power Inc. TLV, Israel), and isopropyl alcohol (Sigma-Aldrich, St Louis, MO, USA) inside an ultrasonic ice bath for 30 min. The ink was sprayed using a manual airbrush on both sides of the membrane mounted on a vacuum hot plate set at 70 °C. Then, the catalyst-coated membrane was hot pressed between two GDLs (Toray carbon paper TGP-030) by metal blocks maintained at 100 °C inside a hydrostatic press (10 MPa) for 6 min. The aluminum mesh (current collectors) was pressed on top of the GDL surface on each side using a lightweight clip. Thus, a very lightweight assembly was achieved, weighing only around 12 g. The MEA was connected at the bottom of the bag, separating the anode chamber from the cathode chamber (#3 in the figure). To create the necessary humidity level (${RH}_{ca}$) of the cathode chamber, a controlled flow of humid air was supplied (#4 in the figure) by a controlled mixture of dry and fully humidified air from a glass water bubbler.

The transferred water through the membrane was measured as a change in the humidity level inside the anode chamber (${RH}_{an}$). This measurement was performed using a microprocessor (Arduino) that reads a humidity-cum-temperature sensor (DHT22) positioned 1 cm away from the anode and the cathode (#6 in the figure). A Potentiostat (Bio-logic^TM^) coupled to the FC’s cathode and anode was used to draw a steady current from the cell and measure the impedance/resistance.

## 3. Results

The results are organized into three sections. Section 3.1 summarizes the effect of the steady-state membrane hydration effect on the current output and its resistance. Section 3.2 shows the dynamic characteristic changes in membrane hydration over time. Section 3.3 presents the model‘s predictions regarding the effect of the MEA and bag configurations on the water content and membrane resistance.

### 3.1. Steady-State Characteristics of the MEA

First, a set of experiments examining the effect of membrane hydration on MEA’s overpotential and resistance were conducted. The setup was conditioned as follows to ensure uniform water concentration across the membrane during the start of each experiment:

Step 1: At the onset of each experiment, the cathode and anode chambers were purged with a flow of dry nitrogen for 30 min to eliminate trapped humidity and air inside the anode chamber.
Step 2: Then, the anode and cathode chambers were maintained at the same selected constant humidity (${RH}_{ca}={RH}_{an}$ = 20–95%) by continuously flowing humid air and hydrogen into the chambers for 30 min.

**Figure 3:**
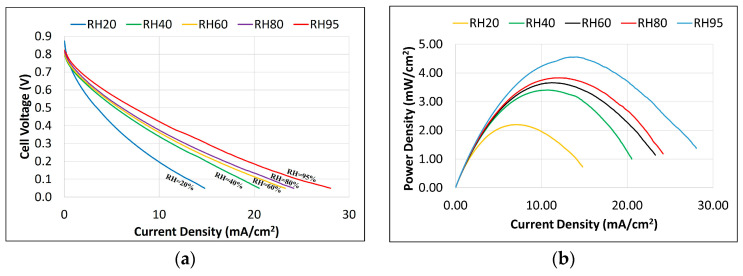
Experimental result of (**a**) polarization test (cell voltage vs. current density) and (**b**) power density at selected humidity levels.

After performing the conditioning step 1 and step 2 as detailed above, polarization tests were conducted where the electrode potential was swept linearly between the open circuit voltage and 0.05 V at a scan rate of 20 mV/minute and the current was measured. The measured current density, cell voltage, and power density at varying humidity levels are shown in Figure 3.

Figure 3 displays an open-circuit voltage (OCV) of approximately 0.8 V with no discernible mass transport limits as the reactant flow was sufficient for the electrochemical reaction and the current was controlled mostly by the ohmic losses. The temperature rise was measured to be negligible (<2 °C). An increase in the maximum current density was evident with an increasing humidity level due to a decrease in the membrane’s ionic resistance, highlighting the critical importance of the hydration level of the membrane in achieving the maximum power output.

As defined above in steps 1 and 2, the setup was also conditioned at varying humidity levels before each impedance test. An electrochemical impedance test measures the high-frequency resistance (HFR) by applying an AC potential (frequency scans: 100 kHz–10 Hz and AC amplitude: 10 mV) to the cell.

**Figure 4:**
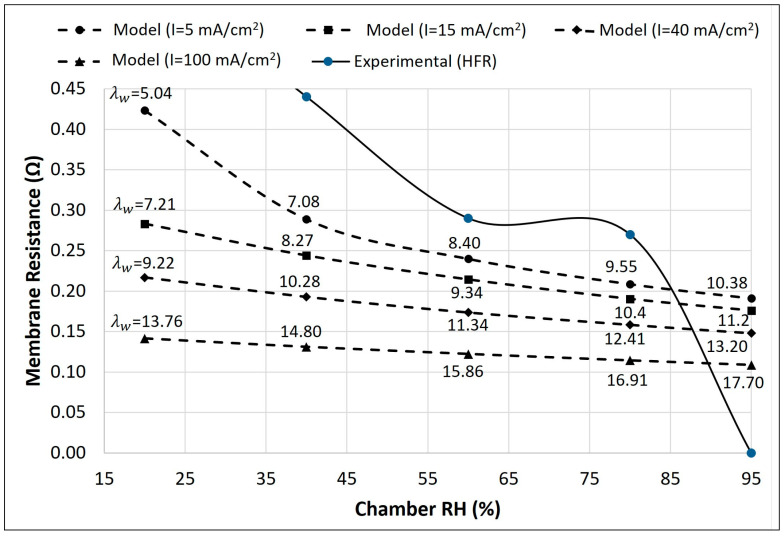
Membrane resistance vs. humidity. The solid line is the experimental results of the impedance test (fixed cathode and anode humidity), and the dashed lines are the model results with varying current densities (fixed cathode and steady-state anode humidity).

HFR measures the ohmic losses in the fuel cell, mainly caused by the ionic resistance of the membrane, effective catalyst layer resistance, and membrane–electrode contact resistance. HFR is commonly used to study the effect of various factors, such as temperature, humidity, gas composition, catalyst loading, membrane thickness, and cell geometry [58,59,60]. The experimental HFR shown in Figure 4 was calculated as presented in Equation (16) to remove any constant resistance (electrical resistance of the wire, etc.) unaffected by humidity changes.

(16) $${HFR}_{mem}={HFR}_{total,\;\;\;\%RH}-{HFR}_{total,\;\;\;RH=95\;\%}$$

Figure 4 displays the experimental membrane resistance (solid line) measurements as a function of membrane humidity and model-predicted (dashed lines) results for membrane resistance at selected current and humidity values. In the model, the membrane resistance was calculated using Equation (15), while the steady-state water content in the membrane ($\lambda_{w}$) was calculated using the parameters in Table 1 at various combinations of current density (*I* = 5, 15, 40, and 100 mA/cm^2^) and cathode humidity (${RH}_{ca}=2$0, 40, 60, 80, and 95%).

The model results indicate a decrease in membrane resistance with an increase in the membrane’s hydration. The model results show a high slope of membrane resistance vs. humidity at a lower current density suggesting that electrochemically produced water is relatively insufficient to contribute any significant hydration to the membrane and the effect of humidity on membrane resistance is more profound. At a high current density (>40 mA/cm^2^), the produced water was significantly higher to maintain the high hydration of the membrane; thus, the effect of the chamber RH becomes minimal in the hydration of the membrane. Both the model and experimental results suggest that a chamber humidity greater than 40% must be maintained to achieve 2–3 times lower membrane resistance compared to dry membrane conditions (RH = 20% and I = 5 mA/cm^2^).

Humidity affects membrane hydration, contact resistance, and mass transport [61,62]. Higher humidity reduces the HFR by increasing membrane hydration and lowering contact resistance due to better membrane–electrode interface wetting. However, high humidity can also increase the HFR due to increased mass transfer resistance, as a high water concentration can hinder the gas diffusion to electrodes by flooding the electrode pores. The experimental results support the model-predicted trend that the MEA’s resistance decreases with increased humidity. At a low humidity (RH < 40%), the membrane and contact resistance was high, which decreased with the increase in RH due to the rise in water content of the membrane and improved wettability of the interface. A detailed interfacial model to calculate the HFR has been published elsewhere and is out of the scope of this work [63,64].

### 3.2. Transient Dynamics of MEA Hydration over Time

After establishing the effect of membrane hydration on its resistance, experimental and numerical methods were used to analyze the transient water transfer dynamics and water content gradient across the membrane over time.

At the start of the experiment, the cathode was equilibrated at the required humidity and the anode chamber was purged with a flow of dry nitrogen for 30 min to eliminate any trapped humidity and air. Then, the anode bag was filled with *V*_0_ = 1000 mL of dry hydrogen, and the current was drawn from the MEA via constant current-controlled discharge. The humidity rise inside the bag was recorded every two seconds for 2 h. Nine experiments were conducted at room temperature (*T* = 20–22 °C) to study the effect of cell current density (*I* = 5, 10, and 15 mA/cm^2^) and cathode humidity (${RH}_{ca}\;$= 20, 50, and 80%) on the humidity inside the anode chamber (${RH}_{an}$).

The numerical simulations were performed by setting a constant cathode humidity (${RH}_{ca}$ = 20, 50, or 80%) and the change in anode chamber humidity concerning the initial value (${RH}_{an}$ = 0 at *t* = 0) was calculated over time (*t*) for a selected current density.

**Figure 5:**
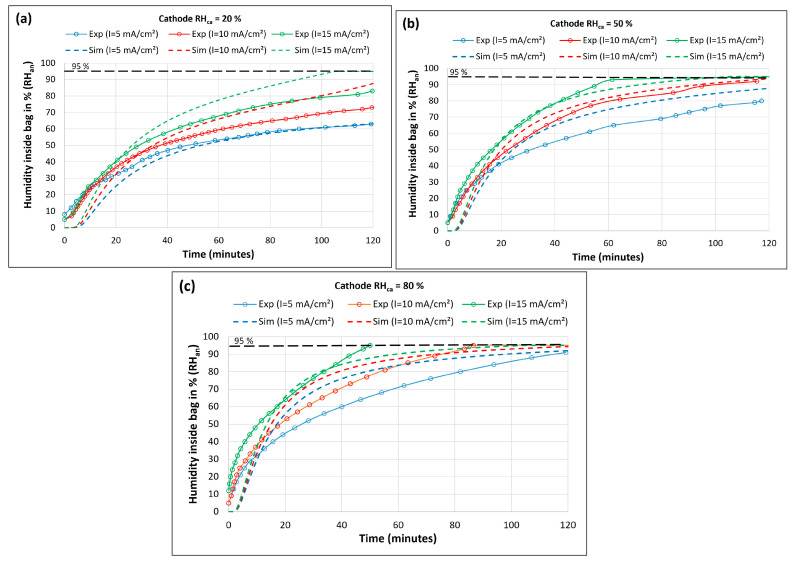
Comparisons between experimental (solid lines with marker) and model (dashed lines) results of humidity in the bag vs. time at varying current density levels at (**a**) *RH_ca_* = 20%, (**b**) *RH_ca_* = 50%, and (**c**) *RH_ca_* = 80%.

Figure 5a–c depicts the humidity inside the bag (${RH}_{an}$) vs. time obtained from the experiment (solid lines with marker) and simulation (dashed lines) at varying current densities (*I*) and cathode humidity levels (${RH}_{ca}$).

The model results agree with the experimentally observed trends that the humidity rise inside the bag increased with an increase in drawn current. More water was produced at the cathode at a higher current density, leading to increased back-diffusion across the membrane from the cathode to the anode. Also, water molecules dragged along with protons from anode to cathode (electro-osmotic drag) increases with increasing current. Our experimental result using a thin membrane suggests that the electro-osmotic drag is lower than back-diffusion at a low current density, resulting in net water flux from the cathode to the anode. This shows that the water coming to the anode side can be trapped/stored in a dead-ended anode chamber for hydrogen and membrane hydration. A thinner membrane is crucial for the effectiveness of such a self-humidification system at a lower current as a thick membrane will reduce both the current and production of water and thus the back-diffusion.

A comparison of the anode humidity at the same current levels in Figure 5a–c shows that the humidity inside the bag increased with an increase in cathode humidity. For instance, a comparison of the experimental humidity results (*RH_an_*) at 15 mA/cm^2^ shows that the time required to reach a 95% RH in the bag was >120, 60, and 50 min for a cathode humidity of 20, 50, and 80%, respectively. Exposure of the cathode to higher environmental humidity increases the water vapor activity at the cathode. It decreases the concentration gradient between the cathode and the ambient environment. Thus, the evaporation of electrochemically produced water from the cathode air is reduced, resulting in increased water mitigation toward the anode. Consequently, this is seen as a faster increase in *RH_an_* inside the bag at higher cathode chamber humidity levels (*RH_ca_*). The hydrogen consumption rates increased with the increase in current density and were determined to be 0.26, 0.44, and 0.76 mL/min at 5, 10, and 15 mA/cm^2^, respectively.

Based on the measured humidity, the amount of water vapor collected inside the bag in 2 h with the base parameters described in Table 1 was estimated to be 0.07 and 0.2 g for current densities of 5 mA/cm^2^ and 15 mA/cm^2^, respectively. The volume of collected water vapor was the same as the volume of the bag (~1 L). It corresponds to a water vapor partial pressure of 0.09 and 0.26 bar for 5 mA/cm^2^ and 15 mA/cm^2^, respectively. The water evaporation rate from the anode to the bag first increased and reached its maximum and then it decreased due to the humidity rise in the bag slowing down the back-diffusion. The maximum was estimated to be 3.27 × 10^−5^ and 5.52 × 10^−5^ g/s for current densities of 5 mA/cm^2^ and 15 mA/cm^2^, respectively.

### 3.3. Effect of MEA and Storage Configurations

**Figure 6:**
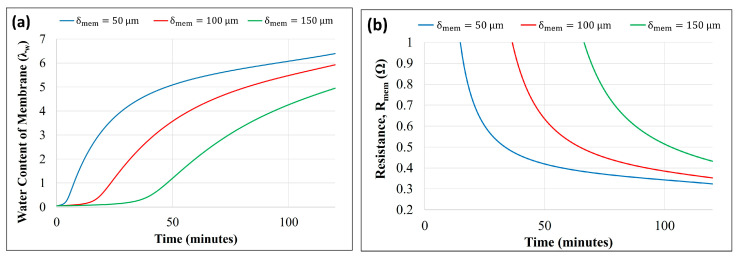
Model prediction of the effect of membrane thickness on (**a**) membrane water content vs. time and (**b**) membrane resistance vs. time.

Figure 6a,b show the effect of varying membrane thicknesses ($\delta_{mem}=50,100,\;and\;150\;\mu m$) on membrane hydration/water content and membrane resistance ($R_{mem}$), respectively, as predicted by the model using the parameters in Table 1.

The dynamic response measurement showed that the fuel cell response time to reach a steady state exceeded 40 min. The accumulation of produced water at the cathode increased with time; thus, the water flux and the relative humidity inside the anode chamber also increased. Increasing the water concentration in the anode chamber reduced the back-diffusion. Initially, the back-diffusion was stronger due to the high water concentration gradient across the membrane, which decreased with time. Thus, the membrane’s water content increased until a steady water balance was established, as shown in Figure 6a. An increase in the water content of the membrane is predicted with a decrease in the membrane’s thickness. A thinner membrane eased the back-diffusion of cathode-produced water to the anode, as diffusion flux is inversely proportional to the thickness. A higher back-diffusion increased the hydration level of the membrane and reduced the ionic resistance, as shown in Figure 6b.

**Figure 7:**
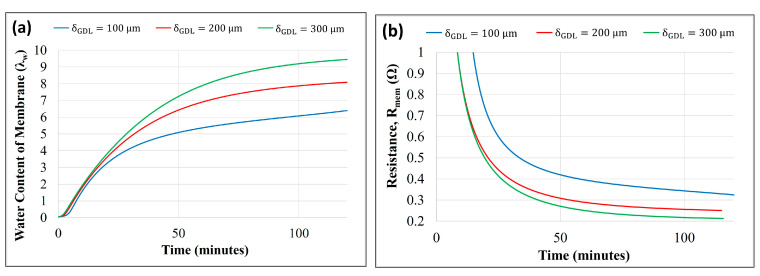
Model prediction of the effect of GDL thickness on (**a**) membrane water content vs. time and (**b**) membrane resistance vs. time.

Figure 7a,b show the effect of varying GDL thicknesses ($\delta_{GDL}^{ca}=\delta_{GDL}^{an}=100,\;200,\;and\;300\;\mu m$) on membrane water content and resistance ($R_{mem}$), respectively.

A higher GDL thickness reduced the water evaporation at the cathode, leading to increased water accumulation, which was subsequently absorbed toward the anode chamber. Thus, a thicker GDL increases the membrane’s back-diffusion and water content. Membrane resistance decreased as the membrane uptake of water increased with an increase in the GDL thickness, as shown in Figure 7b.

The membrane resistance was observed to be more sensitive to membrane thickness than GDL thickness. The resistance of the membrane is a strong function of the water content and is inversely proportional to the thickness. A reduced thickness of the membrane increases the water content and reduces the path resistance. The effect of GDL thickness on electrical resistance was not considered, and only its impact on water transfer across the more often-used membrane and its resistance was modeled.

**Figure 8:**
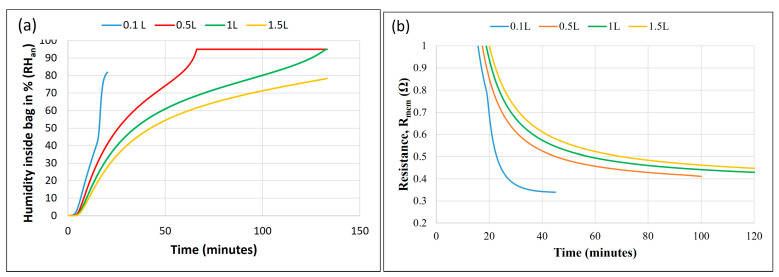
Model prediction of the effect of bag volume on (**a**) anode bag RH (*RH_an_*) vs. time and (**b**) membrane resistance vs. time.

The model predicted the effect of bag volume on humidity at the anode, as shown in Figure 8a, and on membrane resistance, as shown in Figure 8b. The model assumed a maximal humidity of 95% (as measured in the experiments) and hydrogen consumption according to the initial volume.

Figure 8a shows that the relative humidity inside the bag rose faster in a smaller bag volume. The water concentration rose faster in a smaller bag, reducing the water transfer due to back-diffusion. A higher bag volume can accumulate more water flux coming to the anode.

A similar effect of reduced membrane resistance with a decrease in bag volume is shown in Figure 8b. However, a very small bag volume will carry very little hydrogen in the bag and should be selected as per the required duration of operation. Thus, it is proposed to store high humidity in a tiny pouch around the anode only, such that very fast humidification of the anode could be attained, while a large amount of hydrogen could be carried in an isolated hydrogen bag for a longer duration of operation.

## 4. Discussion

Previous studies have focused on FC self-humidification using various methods such as external humidifiers and electrode material mixing [65,66]. Here, we propose a new approach, i.e., a dead-end inflatable hydrogen bag, and identify the crucial variable of such a system. We used a water balance numerical model to explain the transport of the cathode-generated water at an air-breathing open cathode MEA to study the water transport and storage for the design of a self-humidification mechanism. The effect of the Air PEM FC’s component dimensions, such as membrane thickness, GDL thickness, and anode chamber volume for water storage and self-humidification control was investigated.

The model results were validated with experimental system measurements. The model agrees well with the general experimental trend that the water transfer rate increases with increased current density and cathode humidity. The current density and cathode humidity influence the water concentration across the membrane as they cause two competing effects: the produced water can be (1) lost to ambient air (cathode evaporation) or (2) travel to the anode chamber through the membrane. A steady state flux is established when water production at the cathode in an oxygen reduction reaction is balanced by water removal by convection from the anode and cathode. Water vapor transport from the membrane to the gas phases at the anode and cathode is increasing the function of the water concentration in the membrane and the humidity on each side of the MEA. The absorption of cathode-generated water at the membrane can be improved by a suitable selection of the GDL and membrane thicknesses to ensure minimal evaporation at the cathode, e.g., evaporation that can occur by natural air convection, and a net water transfer rate towards the anode is maintained to support self-humidification of its catalytic layer. The model results attained under low humidity conditions show that a thin membrane with a thicker GDL increases the water diffusion flux across the membrane, thus reducing the membrane resistance and improving FC performance. A thinner membrane is preferable for low resistance, but the diffusive gas leakage is also higher through a thinner membrane. A stable voltage and current recorded during the experiments run for 2 h show that the issue of the anode drying out in a closed dead-end anode FC can be resolved by trapping the cathode-produced water at the anode (using a storage bag) during the fuel cell operation.

The water content of the membrane should be maintained at a higher hydration level than the value of $\lambda_{w}=14$ water molecules per membrane sulphonic acid charge site group to maintain high membrane conductivity. An experimental prototype for anode self-humidification was built to demonstrate the feasibility of a low FC power application. However, a high FC power (high current density) could result in additional issues, such as a higher electrode temperature due to increased electrochemical reactions. However, a moderate FC temperature is also reported to increase power output [67,68,69,70,71] due to an increase in reaction activation rate at electrodes (hydrogen oxidation and oxygen reduction) [72], mass transfer rate [67,71], gas diffusivity [73], and membrane conductivity [72]. Moreover, at temperatures above 50 °C, it was also reported to lead to anode dehydration [54] and high activation losses [74]. Thus, a high current density can dehydrate the anode side of the membrane in a continuous flow FC as the electro-osmotic drag is larger at a higher current density and the water dehydration rate of the anode is increased due to the higher temperature. A higher current density can disturb the water transfer balance in the membrane, thus increasing the membrane’s resistance.

A humid hydrogen supply to the anode has been reported to double the power even when operating with a low-humidity cathode air supply [68,70,75]. Thus, the proposed method can trap the produced water to humidify the hydrogen feed stream and maintain high hydration at the anode at all times to prevent membrane dehydration at low ambient humidity and high current density. However, a current density greater than 0.25 A/cm^2^ combined with a higher anode gas humidity can be detrimental to the FC performance and result in flooding at the anode [67,76,77].

Thus, water and heat management are essential to prevent flooding and dehydration, as well as maintaining the operating temperature [78,79,80]. In future work, the presented analytical model may assist in improving the experimental system.

The current study may have implications for various small FC-derived applications. For example, recently, there has been increasing interest in developing lightweight fuel cell-derived blimp drones for surveillance, climate sensors, etc. [81,82,83]. Their power density can be significantly improved by integrating it into lightweight hydrogen storage and on-demand hydrogen production methods at the anode required for electrochemical reactions [84,85,86,87,88,89]. The balloon may serve as a dead-end water and hydrogen storage for the FC’s MEA, thus dramatically improving the device’s endurance and payload.

### Limitations

It is noted that the numerical model oversimplifies the boundary conditions and coupling between mass transfer, thermal transfer, and electrochemical kinetics, which is more significant in an FC stack or at a higher current density and temperatures of the system. A higher current could not be obtained due to a large contact resistance, which requires us to develop a better compression system in the future. Thus, an experimental result at a high current and temperature, along with quantitative results using a more detailed model, remains an issue to be discussed in our future work.

## 5. Conclusions

- A lightweight, inflatable hydrogen-filled bag around the anode is proposed to trap and store the produced water for self-humidification of the anode.
- As demonstrated with an experimentally validated numerical model, the water transport of FC-produced water from the cathode to the anode increases with current density and cathode humidity.
- The power output almost doubles, and membrane resistance is reduced by 2–3 times when a fully hydrated membrane is used compared to a dry membrane.
- The model under equilibrium predicts an increase in membrane resistance by about three-fold with an increase in membrane thickness (50–150 µm) and a decrease of approximately three times with an increase in GDL thickness (100–300 µm).

## Figures and Tables

**Table 1:** Base model parameters.

S. No.	Parameter	Name	Value
1	$A_{mem}$	Area of MEA	4 × 10^−4^ m^2^
2	$\delta_{mem}$	Thickness of membrane	50 μm
3	$\delta_{GDL}^{ca}$	Thickness of cathode GDL	100 μm
4	$\delta_{GDL}^{an}$	Thickness of anode GDL	100 μm
5	*I*	Current density	5 mA/cm^2^
5	${RH}_{ca}$	Cathode chamber humidity	0.2
6	*V*_0_	Bag initial volume	1 L

## Data Availability

Data is contained within the article.
